# Correlates of excessive daytime sleepiness in obstructive sleep apnea: Results from the nationwide SESAR cohort including 34,684 patients

**DOI:** 10.1111/jsr.13690

**Published:** 2022-07-22

**Authors:** Martin Ulander, Jan Hedner, Göran Stillberg, Ola Sunnergren, Ludger Grote

**Affiliations:** ^1^ Department of Biomedical and Clinical Sciences, Faculty of Medicine Linköping University Linköping Sweden; ^2^ Department of Clinical Neurophysiology Linköping University Hospital Linköping Sweden; ^3^ Department Pulmonary Medicine Sahlgrenska University Hospital Gothenburg Sweden; ^4^ Centre for Sleep and Wake Disorders, Sahlgrenska Academy Gothenburg University Gothenburg Sweden; ^5^ Sleep Clinic, Capio Läkargruppen Örebro Sweden; ^6^ Department of Otorhinolaryngology, Region Jönköping County, and Department of Biomedical and Clinical Sciences Linköping University Linköping Sweden

**Keywords:** registry‐based study, sleep‐related breathing disorders

## Abstract

Excessive daytime sleepiness (EDS) is a hallmark symptom in obstructive sleep apnea (OSA). It is commonly eliminated by obstructive sleep apnea therapy and constitutes a major treatment indication. This study aimed to identify determinants of excessive daytime sleepiness by the Epworth Sleepiness Scale (ESS) scores in the large, representative national obstructive sleep apnea patient cohort of the Swedish Sleep Apnea Registry (SESAR, www.sesar.se). Data from 34,684 patients with obstructive sleep apnea recruited at 23 sites (33% females, mean age 55.7 ± 13.7 years, BMI 30.2 ± 6.3 kg/m^2^, AHI 29.1 ± 22.3, and ODI 24.9 ± 21.4 events/h) had a mean ESS score in the mild to moderate excessive daytime sleepiness range (9.7 ± 4.9). The proportion of patients with excessive daytime sleepiness was 41.4% in men and 44.6% in women. Independent predictors of excessive daytime sleepiness included gender, age, and hypoxic markers (high ODI and low mean saturation). Univariate and multivariate analyses were used to identify significant predictors for the ESS score and for excessive daytime sleepiness (ESS ≥10) amongst anthropometric factors, sleep apnea frequency (apnea‐hypopnea index (AHI)), markers of intermittent hypoxia (oxygen desaturation index (ODI), mean saturation (mSaO_2_)), as well as prevalent comorbidities. Depression was associated with higher ESS scores and hypertension/atrial fibrillation with lower scores. The oxygen desaturation index provided a stronger predictor of excessive daytime sleepiness than the apnea‐hypopnea index. The severity of obstructive sleep apnea, captured as the apnea‐hypopnea index, was only weakly associated with daytime sleepiness in this representative obstructive sleep apnea patient cohort. Age had different effects in men and women.The impact of obstructive sleep apnea in a wider patient related perspective needs to be determined after the inclusion of factors other than the apnea‐hypopnea index.

## 1 INTRODUCTION

Obstructive sleep apnea (OSA) is characterised by repetitive episodes of total (i.e., apneic) or partial (i.e., hypopneic) mechanical obstruction of the upper airway. These events, which lead to oxygen desaturation and arousal from sleep, have been linked to negative short and long‐term health consequences and sleepiness (Knauert et al., 2015). The severity of obstructive sleep apnea is conventionally quantified by the number of apneas, hypopneas, and desaturations per hour of sleep, and is expressed in terms of the apnea‐hypopnea index (AHI) and the oxygen desaturation index (ODI). However, the prevalence of obstructive sleep apnea depends on the diagnostic criteria applied for classification of the apneas, hypopneas, and desaturations but is reported to be 9%–38% of the general population (Senaratna et al., 2017).

Excessive daytime sleepiness (EDS) is a major limiting symptom in obstructive sleep apnea, which affects daytime functioning and quality of life. Sleepiness, which has been linked to an increased risk of accidents, also provides a significant health economic burden (Léger & Stepnowsky, 2020). Furthermore, sleepy men with obstructive sleep apnea had higher levels of C‐reactive protein, indicating systemic inflammation, than non‐sleepy men with obstructive sleep apnea, even after controlling for confounders (Andaku et al., 2015), and Vgontzas et al. (1997) found higher levels of circulating pro‐inflammatory cytokines in patients with sleep disorders associated with excessive daytime sleepiness compared with normal controls. Obstructive sleep apnea with excessive daytime sleepiness might therefore be associated with a higher risk of systemic diseases than obstructive sleep apnea without excessive daytime sleepiness. Importantly, excessive daytime sleepiness in obstructive sleep apnea may effectively be eliminated by obstructive sleep apnea therapy. The functional association between obstructive sleep apnea severity and sleepiness is complex. Some patients with severe obstructive sleep apnea report only marginal daytime sleepiness, while others may complain of debilitating excessive daytime sleepiness. Factors linked to excessive daytime sleepiness in patients with obstructive sleep apnea include the frequency of respiratory events in rapid eye movement (REM) sleep (Gabryelska & Białasiewicz, 2020), gender (Chervin, 2000), younger age (Budhiraja et al., 2017), higher body mass index (BMI) (Slater et al., 2013), and comorbid conditions (Bixler et al., 2005; Kapur et al., 2005; Martynowicz et al., 2017). Most studies in this field are small and of a single‐centre design with the risk of a limited number of covariates and/or patient bias.

The Swedish Sleep Apnea Registry (SESAR), which was started in 2010, has reached a coverage of approximately 60% of all Swedish centres providing a sleep medicine service to patients with suspected obstructive sleep apnea. SESAR collects demographic data, clinical data (e.g., comorbidities and cardiorespiratory recording data), and the results from the sleep test during the initial diagnostic work‐up. The aim of our study was to identify determinants of excessive daytime sleepiness in the national SESAR cohort of patients with newly diagnosed obstructive sleep apnea.

## 2 METHODS

The SESAR registry collects data on anthropometrics, comorbid disorders, sleep study results, and patient outcome measures at different time points during the clincial management cycle of diagnosis, treatment, and follow up. By the end of 2018, data from 34,684 unselected patients visits had been manually entered at 23 sleep centres across the country. This corresponds to a national coverage exceeding 60% of all diagnostic procedures performed in Sweden in 2021. In order to be included in the registry, patients are expected to have undergone either a cardioresporatory polygraphy or a polysomnography indicating obstructive sleep apnea, and a clinical examination by a physician who formally diagnoses them, but they do not need to have started any treatment.

Clinically relevant data in the registry are publicly available at a group level on the registry web page (www.sesar.se/statistik). Via an interactive function the web page visualises between‐centre comparisons of patient characteristics, waiting times, and treatment outcomes (e.g. adherence to positive airway pressure (PAP), mandibular advancement devices (MAD) or the proportion of centres who initiated weight reduction therapy in obese obstructive sleep apnea patients). Patients and health care personnel are encouraged to use the interface to monitor and stimulate improvement in routine health care.

### 2.1 The patient sample

All subjects registered in the SESAR from April 2010 until December 2019 were included. The main inclusion criteria in the quality registry is the clinical diagnosis of OSA (ICD10 G47.3) according to ICSD 2 criteria (American Academy of Sleep Medicine, 2005). Due to a lack of centralised data on the number of obstructive sleep apnea cases in Sweden, the best possible estimate of coverage of the SESAR is around 60% according to data from the National Board of Health and welfare. The only exclusion criterion applied in our analysis was missing ESS data (<10% of reported patients). Patients with sleep related hypoventilation without obstructive sleep apnea are reported in a separate quality registry (www.swedevox.se). Patients with predominately central sleep apnea are not targeted in the SESAR registry.

### 2.2 Assessment of sleep disordered breathing

Sleep apnea was assessed, in more than 99% of cases, by cardiorespiratory polygraphy. Data were analysed according to AASM diagnostic criteria (Berry et al., 2012; Iber et al., 2007). Hypopneas were scored with a concomitant 4% desaturation in the majority of centres. National guidelines for scoring obstructive sleep apnea were first defined in 2018 (Hedner et al., 2018). Data were manually edited and entered into the registry via a web‐based platform (www.sesar.se).

Hypopneas associated with arousals but without desaturations were generally not recognised or counted during cardiorespiratory polygraphy. Therefore, a small number of patients with this respiratory pattern, as well as some patients with strictly positional obstructive sleep apnea, may have received a diagnosis of sleep apnea despite an apnea‐hypopnea index below 5 events/h and thereby were entered into the registry.

### 2.3 Assessment of daytime sleepiness

Daytime sleepiness was assessed using the Epworth Sleepiness Scale score (ESS) (Johns, 1991). The ESS lists eight everyday situations and the probability of falling asleep using a four‐point Likert‐type scale ranging from 0 (no chance of dozing) to 3 (high chance of dozing). Individual item scores are summed to a total score ranging from 0 to 24, with high values indicating more severe daytime sleepiness. Patients completed the ESS when they were entered into the registry at the time of diagnosis and before any active treatment was applied. The data for the current analysis were obtained at the end of the diagnostic procedure.

### 2.4 Comorbidities

The SESAR registry mirrors the prevalence of major relevant comorbidities associated with obstructive sleep apnea including cardiovascular, metabolic, and psychiatric disease. The scope of the comorbidities recorded has been expanded gradually over time. In particular, conditions such as atrial fibrillation and a distinction between asthma and COPD are conditions more recently added.

### 2.5 Statistical analyses

Statistics were performed in R, version 3.6, with packages lme4 (regression models), mice (multiple imputation), and DHARMa (regression model diagnostics). Linear and logistic regression were used to identify correlates of daytime sleepiness. Daytime sleepiness (ESS) was used as the dependent variable, both in linear regression and in logistic regression models. For the purposes of the logistic regression, two cutoff points were used: in one set of models EDS was defined as ESS≥10, and in the other set of models EDS was defined as ESS≥15. The choice of independent variables to add to the regression models was based on a combination of univariate regression models, where each variable was used as a predictor of ESS, and theoretical considerations based on previous findings from the literature (see Table e1, online supplement). The regression models were first generated as fully pooled models (i.e., all subjects were pooled without regard to what centre they were treated at), and then, in a second step, as two‐level hierarchical models (with patients in one level, and centres in a second level) in order to accommodate for local differences in access to obstructive sleep apnea treatment, diagnostic procedures etc. Analyses were performed both on complete cases and after handling missing data with multiple imputation with chained equations (van Buuren, 2012). As adding both the apnea‐hypopnea index and the oxygen desaturation index to the regression models caused issues with multicollinearity, the apnea‐hypopnea index was dropped as a predictor from the final models. The choice to drop the apnea‐hypopnea index over the oxygen desaturation index was based on comparing Akaike's information criterion (Akaike, 1974) between a model retaining the apnea‐hypopnea index but not the oxygen desaturation index, and a model retaining oxygen desaturation index but not apnea‐hypopnea index.

## 3 RESULTS

### 3.1 Clinical data of the national patient cohort

Background clinical and demographic data for all subjects in relation to the severity of EDS are presented in Table 1. Demographic and clinical data for individual centres are presented in Table e2 (online supplement).The main differences between reporting sleep centres were related to disease severity (i.e., AHI and ODI means, ranging from 17–37/h and 16–35/h, respectively), and smoking habits (current smokers 9.3 to 42.9%) (Table e2, online supplement). The proportion of patients with excessive daytime sleepiness was 41.4% in men and 44.6% in women when using a cutoff of ESS≥10, and 16.9% and 24.0%, respectively, when using a cutoff of ESS≥15. The mean ESS reported by the participating centres in the study varied between 8.5 and 11.3, suggesting a considerable degree of sleepiness among the reported patients. A sensitivity analysis showed no clinically relevant change of mean ESS score over time (year of registration).

**TABLE 1:** Background demographic and clinical data of the sample

Gender (%) M/F	67.2/32.9
Age (years) M ± SD	55.7 ± 13.7
ESS M ± SD	9.7 ± 4.9
ESS ≥10 (%)	42.5
ESS ≥15 (%)	17.7
AHI (h^−1^) M ± SD	29.1 ± 22.3
ODI (h^−1^) M ± SD	24.9 ± 21.4
Average saturation (%) M ± SD	92.2 ± 2.6
BMI (kg/m^2^) M ± SD	30.2 ± 6.3
Smoking (%)	
Not at all	52.7
Daily	10.8
Comorbidities (%)	
Hypertension	48.3
Coronary heart disease	10.9
Cerebrovascular disease	5.3
Atrial fibrillation	9.9
Depression	14.5
Heart failure	5.0
Diabetes	14.4
COPD/Asthma	12.6
Two or more comorbid conditions	28.8

### 3.2 Factors influencing the **ESS** score

**FIGURE 1:**
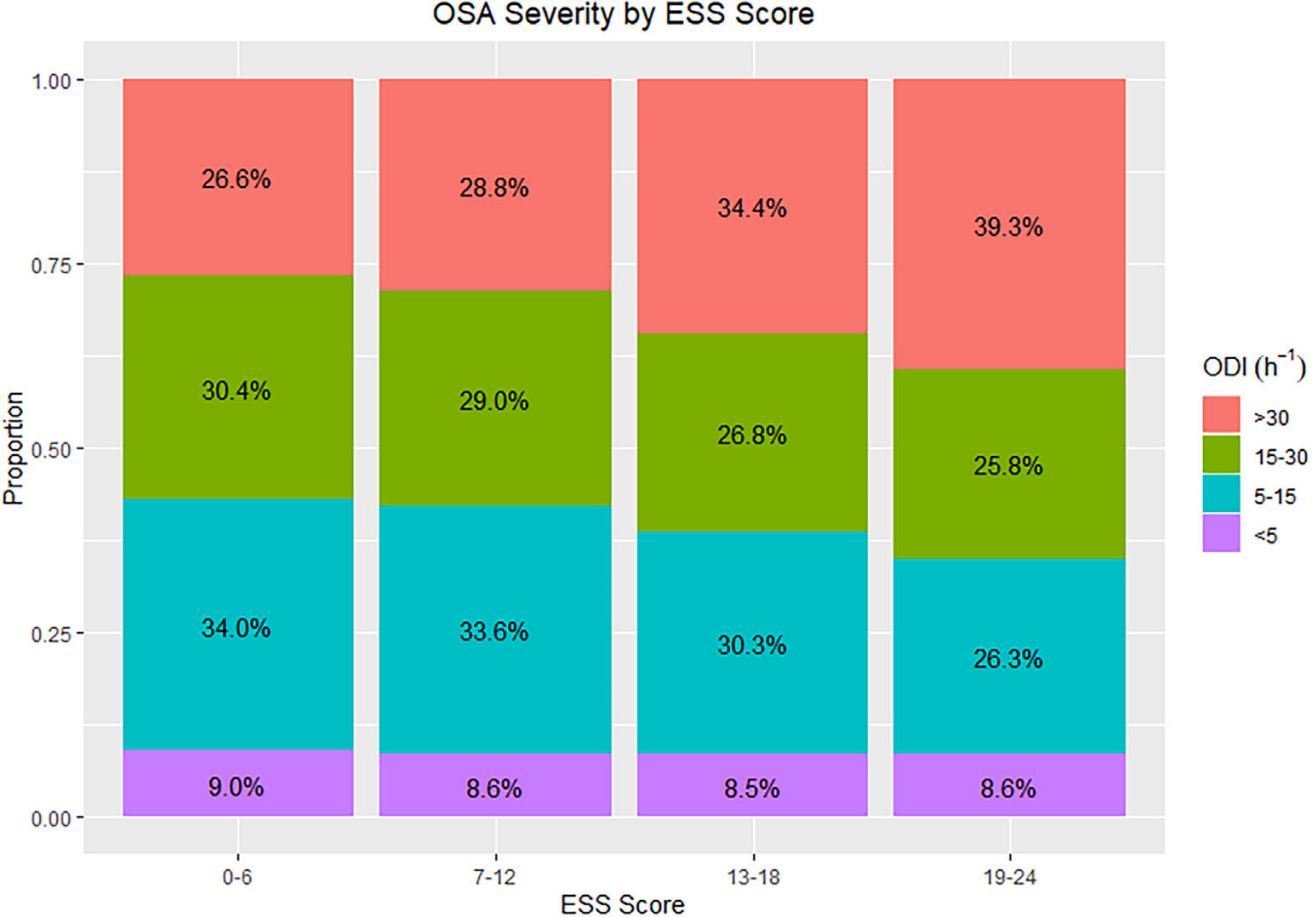
Distribution of the severity of obstructive sleep apnea as defined by ODI among subjects with different ESS scores. There is a greater proportion of subjects with severe obstructive sleep apnea among the more sleepy subjects

**FIGURE 2:**
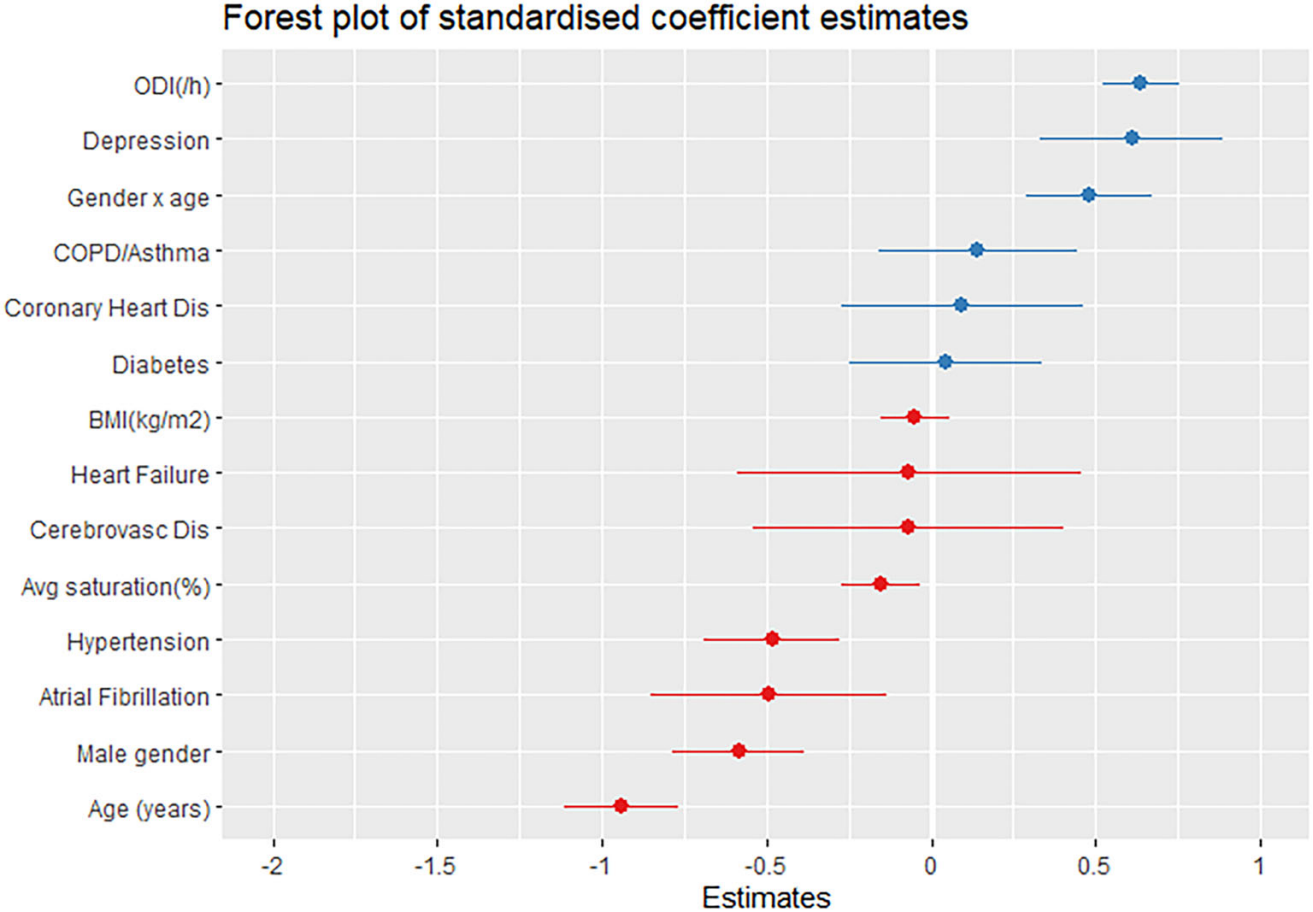
Logistic regression coefficients presented as forest plot. The horizontal bars represent 95% confidence interval for the odds ratios

The number of patients suffering from significant daytime sleepiness (ESS score 10 and above) was weakly associated with more severe obstructive sleep apnea (Figure 1). The univariate regression models are presented in Table 2. The results from the multivariate linear regression analyses are presented in Table 3. In univariate, completely pooled analyses, female gender was associated with a higher degree of self reported sleepiness. Moreover, a higher severity index for the apnea‐hypopnea index, the oxygen desaturation index, the mean oxygen saturation, a higher BMI, younger age as well as a diagnosis of depression were all associated with more sleepiness. Conversely, comorbidities including hypertension, coronary heart disease, cerebrovascular disease, or atrial fibrillation were associated with less sleepiness. In the multiple linear regression models ODI, gender, age, hypertension, atrial fibrillation, and depression were significant correlates to daytime sleepiness in multilevel models (Table 3). There was also an interaction between age and gender suggesting that older age predicted sleepiness more strongly in women compared with in men. The mean oxygen saturation was also related to daytime sleepiness in the multilevel models (Figure 2).

**TABLE 2:** Significant correlates of daytime sleepiness in univariate regression models

Variable	Linear (β ± SE)	Logistic (OR, 95% CI)
Male gender	**−0.264 ± 0.059**	**0.879 (0.838–0.922)**
AHI (h^−1^)	**0.023 ± 0.001**	**1.007 (1.006–1.008)**
ODI (h^−1^)	**9.037 ± 0.043**	**1.009 (1.008–1.010)**
Average saturation (%)	**−0.109 ± 0.011**	**0.967 (0.958–0.975)**
BMI (kg m^−2^)	**0.0638 ± 0.004**	**1.021 (1.017–1.024)**
Age (years)	**−0.050 ± 0.002**	**0.983 (0.981–0.985)**
Diabetes	**−0.449 ± 0.089**	**0.834 (0.776–0.897)**
Heart failure	**−0.603 ± 0.147**	**0.786 (0.697–0.886)**
COPD/Asthma	**0.301 ± 0.122**	**1.110 (1.007–1.224)**
Coronary heart disease	**−0.994 ± 0.102**	**0.682 (0.626–0.741)**
Depression	**0.781 ± 0.091**	**1.328 (1.235–1.428)**
Cerebrovascular disease	**−0.789 ± 0.143**	**0.735 (0.653–0.826)**
Atrial fibrillation	**−1.149 ± 0.133**	**0.662 (0.591–0.740)**
Hypertension	**−1.015 ± 0.061**	**0.703 (0.669–0.738)**
Smoking^a^	**0.627 ± 0.097**	**1.241 (1.149–1.342)**

^a^ Not at all versus daily.

**TABLE 3:** Results from multivariate linear regression models

	Fully pooled model			Multilevel model (complete cases)			Multiply imputed multilevel model		
Correlate	Coefficient estimate (β)	Standard error	Student's *t*	Coefficient estimate (β)	Standard error	Student's *t*	Coefficient estimate (β)	Standard error	Student's *t*
Male gender^a^	**−2.84**	**0.44**	**−6.51**	**−2.72**	**0.43**	**−6.28**	**−2.45**	**0.26**	**−9.49**
Age (years)	**−0.07**	**0.01**	**−10.54**	**−0.07**	**0.01**	**−10.42**	**−0.07**	**0.00**	**−16.80**
ODI	**0.03**	**0.00**	**11.42**	**0.03**	**0.00**	**10.30**	**0.03**	**0.00**	**16.14**
Average saturation	−0.03	0.02	−1.28	**−0.06**	**0.02**	**−2.37**	**−0.07**	**0.01**	**−4.89**
BMI	−0.01	0.01	−0.61	−0.01	0.01	−1.08	0.00	0.01	0.44
Hypertension	**−0.47**	**0.11**	**−4.31**	**−0.47**	**0.11**	**−4.32**	**−0.56**	**0.07**	**−7.80**
Coronary heart disease	0.07	0.19	0.38	0.04	0.19	0.21	−0.12	0.14	−0.88
Cerebrovascular disease	−0.15	0.25	−0.61	−0.08	0.25	−0.33	−0.00	0.14	−0.01
Atrial fibrillation	−0.36	0.19	−1.90	**−0.47**	**0.19**	**−2.49**	**−0.39**	**0.10**	**−3.75**
Depression	**0.76**	**0.15**	**5.10**	**0.64**	**0.15**	**4.35**	**0.53**	**0.10**	**5.46**
Heart failure	0.05	0.28	0.18	−0.05	0.28	−0.17	−0.04	0.12	−0.30
Diabetes	0.17	0.16	1.07	0.09	0.15	0.61	−0.04	0.09	−0.44
COPD/Asthma	0.29	0.16	1.83	0.14	0.16	0.92	0.23	0.14	1.66
Daily smoking	0.18	0.16	1.08	0.16	0.16	1.02	0.09	0.09	1.83
Gender × Age^a^	**0.04**	**0.01**	**5.19**	**0.04**	**0.01**	**5.06**	**0.04**	**0.00**	**8.01**

Correlates of ESS scores in patients with obstructive sleep apnea. Bold‐faced lines are significant (*p* < 0.05).

^a^ The effect of age on sleepiness is stronger in women than in men.

### 3.3 Analysis of factors associated with excessive daytime sleepiness

In the univariate logistic regression models, a higher probability of excessive daytime sleepiness was associated with female gender, high BMI, young age, low mean oxygen saturation, high apnea‐hypopnea index and oxygen desaturation index, daily smoking (compared with non‐smoking), absence of atrial fibrillation, coronary heart disease, cerebrovascular disease, diabetes, heart failure, and the presence of COPD/asthma or depression. Results from the logistic regression are presented in Table 4 (ESS≥10) and in Table e3 (ESS≥15). In general, the findings matched the findings of the linear regression models. However, daily smoking (compared with not smoking at all) was significantly associated with EDS when a higher ESS cutoff point was used (smokers were slightly more likely to be sleepy).

**TABLE 4:** Results from multiple logistic regression models for excessive daytime sleepiness

	Fully pooled model			Multilevel model (complete cases)			Multiply imputed multilevel model		
	Coefficient estimate (OR)	Standard error	OR 95% CI	Coefficient estimate (OR)	Standard error	OR 95% CI	Coefficient estimate (OR)	Standard error	OR 95% CI
Male gender	**−0.96 (0.39)**	**0.19**	**0.27–0.56**	**−0.27(0.77)**	**0.05**	**0.70–0.84**	**−0.20 (0.81)**	**0.03**	**0.77–0.86**
Age	**−0.02 (0.98)**	**0.00**	**0.97–0.98**	**−0.33 (0.72)**	**0.04**	**0.66–0.78**	**−0.32 (0.73)**	**0.03**	**0.69–0.76**
ODI	**0.01 (1.01)**	**0.00**	**1.01–1.01**	**0.23 (1.26)**	**0.03**	**1.20–1.33**	**0.19 (1.21)**	**0.02**	**1.17–1.24**
Average saturation	−0.00 (1.00)	0.01	0.98–1.02	−0.03 (0.97)	0.03	0.92–1.02	**−0.06 (0.94)**	**0.02**	**0.91–0.97**
BMI	−0.01 (1.00)	0.00	0.99–1.00	**−0.05 (0.95)**	**0.02**	**0.91–1.00**	−0.01 (1.00)	0.01	0.97–1.02
Hypertension	**−0.04 (0.85)**	**0.01**	**0.78–0.94**	**−0.16 (0.85)**	**0.05**	**0.78–0.93**	**−0.20 (0.82)**	**0.03**	**0.77–0.87**
Coronary heart disease	0.07 (1.07)	0.08	0.91–1.26	0.06 (1.06)	0.08	0.90–1.25	−0.07 (0.93)	0.05	0.85–1.02
Cerebrovascular disease	−0.05 (0.95)	0.11	0.77–1.17	−0.03 (0.97)	0.11	0.79–1.21	−0.01 (0.99)	0.07	0.87–1.14
Atrial fibrillation	−0.08 (0.92)	0.08	0.79–1.08	−0.12 (0.89)	0.08	0.76–1.05	**−0.22 (**0.80)	**0.07**	**0.68–0.94**
Depression	**0.25 (1.28)**	**0.06**	**1.13–1.45**	**0.22 (1.24)**	**0.06**	**1.10–1.40**	**0.19 (1.2)**	**0.04**	**1.12–1.31**
Heart failure	−0.00 (1.00)	0.12	0.79–1.26	−0.04 (0.96)	0.12	0.76–1.22	0.00 (1.00)	0.08	0.85–1.18
Diabetes	0.06 (1.06)	0.07	0.93–1.21	0.04 (1.04)	0.07	0.91–1.18	0.01 (1.01)	0.04	0.93–1.09
COPD/Asthma	**0.15 (1.16)**	**0.07**	**1.02–1.32**	0.10 (1.11)	0.07	0.97–1.26	0.09 (1.09)	0.05	0.98–1.21
Daily smoking	0.06 (1.06)	0.07	0.92–1.21	0.06 (1.06)	0.07	0.92–1.21	0.07 (1.07)	0.04	0.99–1.16
Gender × Age^a^	**0.01 (1.01)**	**0.00**	**1.01–1.02**	**0.16 (1.18)**	**0.04**	**1.08–1.28**	**0.17 (1.18)**	**0.03**	**1.12–1.25**

Correlates of excessive daytime sleepiness in multivariate logistic regression models. Bold‐faced lines are significant (*p* < 0.05). Continuous predictors are standardised.

Abbreviations: CI, confidence interval; OR, odds ratio.

^a^ The effect of age on sleepiness is stronger in women than in men.

## 4 DISCUSSION

### 4.1 Main findings of the study

This is a large, multicentre national cohort study addressing the factors associated with daytime sleepiness in patients with obstructive sleep apnea. Traditional disease severity indices such as the apnea‐hypopnea index and the oxygen desaturation index were both related to daytime sleepiness but the associations were weak. Factors such as age, gender, and comorbidity also affected daytime sleepiness in patients with obstructive sleep apnea even after including an extensive number of variables. There was a substantial amount of unexplained variance in the final statistical model and while some comorbidities such as depression were associated with a higher degree of daytime sleepiness, others such as hypertension were consistently related to less EDS. The BMI, which has been associated with daytime sleepiness in the general population (Bixler et al., 2005) as well as in clinical obstructive sleep apnea cohorts (Garbarino et al., 2018; Shao et al., 2019), was not significantly associated with daytime sleepiness in our study in fully adjusted models, although it was associated in univariate models. In addition, male gender, which previously has been linked to EDS (Garbarino et al., 2018), was associated with less sleepiness in the current analysis when compared with females. These differences in the results may reflect the heterogeneity in the referral pattern to sleep centres between countries. Indeed, our cohort had a moderately increased BMI of around 30 kg/m^2^, which was substantially lower than reported in other large patient cohorts such as the ESADA (Hedner et al., 2011).

While EDS is a common symptom in obstructive sleep apnea, and the relationship between obstructive sleep apnea severity and EDS severity is relatively weak, different studies have reported somewhat different results with regard to what the associated covariates are. Sleepiness is one of the main motivating factors for treatment in obstructive sleep apnea, given the risk of sleepiness associated occupational and traffic accidents. There are several possible reasons for this apparently weak association. Gender and age interact with regard to EDS, meaning that different studies with different age and gender distribution may report different findings if these interactions are not taken into account. Factors affecting daytime sleepiness in obstructive sleep apnea patient cohorts may do so through different mechanisms: apart from a physiological association (i.e., a comorbidity such as hypertension may be asssociated with EDS via a shared biomedical mechanism), there is also the possibility that certain groups of patients (e.g., men or patients with known pre‐existing conditions) are more easily referred to a sleep study, thereby getting an obstructive sleep apnea diagnosis. Other patients may need to report more symptoms (e.g., EDS) in order to be referred. It is also worth noting that the present measurements of both obstructive sleep apnea and EDS severity (e.g., AHI and ESS, respectively), have been criticised for not measuring what is important.

### 4.2 Gender effects on OSA/EDS

Several studies on obstructive sleep apnea related excessive daytime sleepiness have reported less sleepiness in women compared with men. A questionnaire based study in a community sample (Appleton et al., 2018) showed a stronger correlation between the excessive daytime sleepiness and self‐reported diagnosed obstructive sleep apnea in women than in men. Moreover, men with obstructive sleep apnea were more likely to end up in a moderately sleepy cluster compared with women who were more likely to be in the extensively sleepy cluster (Morris et al., 2021). The higher degree of sleepiness in women in our cohort was no longer associated with the BMI when variables other than the oxygen desaturation index were accounted for. It can be speculated that part of the daytime sleepiness associated with BMI in previously reported population samples may be accounted for by undiagnosed obstructive sleep apnea. Further, referring physicians may perceive obstructive sleep apnea more as a “male disease”, thereby systematically neglecting symptoms of daytime sleepiness in women. Lindberg et al. (2017) reported that a larger proportion of men with symptoms of obstructive sleep apnea (snoring and EDS) received an obstructive sleep apnea diagnosis and subsequent treatment, compared with women with similar symptoms. In our study, there was also a significant interaction between gender and age, meaning that age affected excessive daytime sleepiness differently in men and women.

### 4.3 OSA/EDS and cardiovasular disease

The clinical significance of cardiovascular disease in obstructive sleep apnea is emphasised in international guidelines for atrial fibrillation and hypertension (Hindricks et al., 2021; Williams et al., 2018). Hence, it is likely that more referrals to sleep laboratories would be expected among patients with cardiovascular disease although they lack overt symptoms of sleepiness. Previous research provides somewhat conflicting results with regard to the association between hypertension and excessive daytime sleepiness in obstructive sleep apnea patients. Martynowicz et al. (2017) found less sleepiness among hypertensive obstructive sleep apnea patients compared with their normotensive counterparts, while Feng et al. (2012) found that ESS was positively correlated with blood pressure. However, Martynowicz studied patients with previously known hypertension while Feng et al. (2012) evaluated consecutive patients referred for sleep studies at several hospitals in China. Our study compares better with that of Martynowicz et al, considering that it addresses patients with previously diagnosed hypertension. Interestingly, less pronounced sleepiness in patients with hypertension has been attributed to sympathetic activation which is strongly associated with sleep apnea (Seravalle et al., 2018). A similar mechanistic explanation has been proposed for the apparent lack of daytime sleepiness in heart failure patients with sleep apnea (Pak et al., 2019).

### 4.4 EDS and sleep apnea severity

The correlation between severity of the obstructive sleep apnea condition, conventionally expressed by the AHI and daytime sleepiness was weak in our study in line with previously well established data (e.g., Kingshott et al., 1995). This notion may have considerable consequences as, for instance, the apnea‐hypopnea index is given a central role for instance when establishing fitness to drive according to EU directives (European Union directive 2014/85/EU). In this context regression models based on the oxygen desaturation index, rather than the apnea‐hypopnea index, might be prefered as they had better goodness‐of‐fit statistics. The oxygen desaturation index and the apnea‐hypopnea index were fairly strongly correlated in this patient database, a finding most likely explained by the high proportion of patients that were diagnosed with cardiorespiratory polygraphy rather than with polysomnography. Hypopneas with arousals but only a marginal effect on blood oxygen saturation have been missed. Still, the better fit for ODI‐based models is in line with other studies with similar design but different outcomes (Tkacova et al., 2014), as well as studies with different design but with the same outcome (i.e., daytime sleepiness; Kainulainen et al., 2019).

### 4.5 Methods to assess daytime sleepiness

The ESS score is used in both clinical and research studies. The questionnaire is well validated and easy to use but the process used to generate the items is poorly described, and some questions contain ambiguous wordings. While some studies only found a moderate correlation between the ESS and other funtional measures of daytime sleepiness such as the multiple sleep latency test or the maintenance of wakefulness test (e.g., Chervin et al., 1997), other studies could not demonstrate a correlation (Chervin & Aldrich, 1999). In fact, the associations between the ESS score and the degree of obstructive sleep apnea are usually quite poor (Gottlieb et al., 1999). Sensitivity and specificity to detect obstructive sleep apnea cases with an elevated risk for traffic accidents was significant only at very high ESS scores exceeding 15 (Karimi et al., 2015). Despite the fundamental criticism of the ESS in the field, the scale is internationally widely applied most likely due to the fact that no better instrument is available in the clinical routine. This is evidenced by the high completeness rate of the ESS data in our national study sample reflecting daily medical care.

### 4.6 Strength and limitations of the study

The strengths of the current study include the large sample size that represents a major portion of consecutive patients referred for evaluation of obstructive sleep apnea in Sweden and thereby generating a nationally representative cohort. Furthermore, this was a multicentre study that included most centres in Sweden that diagnose patients with obstructive sleep apnea. Yet another strength is the similarity of findings in several different statistical models (i.e., both linear and logistic models, with both pooled data from all centres and mixed‐effect models, and both complete case analyses and multiple imputation of missing data) with regard to what factors are correlated and the direction of the correlations. This indicates that the findings are fairly robust. Indeed, our results remain largely unchanged after imputation of missing data. Another strength was the uniformity in sleep diagnostic equipment used in Sweden. Almost all diagnostic devices use similar oximetry technology. This supports the validity of the findings on hypoxic markers as predictors of excessive daytime sleepiness (mean overnight saturation and ODI). Finally, the data quality in the SESAR registry is very high. Missing ESS data were below 10% in the total sample and a validation study using in part data of the SESAR registry found a more than 95% congruency between data found in the registry and the medical files (Ekström et al., 2021).

The main weakness of the study is the observational nature of the data, the use of cardiorespiratory polygraphy rather than polysomnography to diagnose obstructive sleep apnea in most cases, the use of the ESS as the only method to assess daytime sleepiness, and the fact that we are restricted with respect to what correlates of daytime sleepiness we can study and by what variables we have available in the SESAR dataset. For example, data on socio‐economic status and education, sleep length and circadian timing, shift work, or physcial excerise levels are unknown. However, clinical quality registries need to be limited in the number of variables to be reported in order to achieve a high coverage of patients. Further, some covariates have been added over time (mainly some of the comorbidities), and are therefore missing in patients' data entered soon after the start of the registry. However, multiple imputation is considered a valid method to deal with missing data, and preferable to many other commonly used methods (van Buuren, 2012). While cardiorespiratory polygraphy is used routinely in clinical practice in Sweden, the golden standard is polysomnography. Ideally, similar studies should be performed in countries where polysomnography is used as the routine examination for obstructive sleep apnea, rather than where it is mostly used in selected cases where a high clinical suspicion of obstructive sleep apnea remains despite a negative cardiorespiratory polygraphy. Regarding measurements of daytime sleepiness, while the ESS is a simple and clinically widely used tool, it is only a subjective scale. Using objective measurements, such as the Multiple Sleep Latency Test might add more information, but would be unfeasible in such a large cohort. With large datasets collected in several sites, there is also always a risk that some data for individual patients may be erroneously entered by the reporting centres. This is a general reservation in epidemiologic research using registry data, but given the size of the cohort, it is unlikely that individual errors would affect the findings to a major extent.

In conclusion, this large multicentre study of daytime sleepiness in obstructive sleep apnea has demonstrated that traditional disease severity indices such as the apnea‐hypopnea index and the oxygen desaturation index are linked to daytime sleepiness but the association is rather weak. Further research should focus on including novel potential disease severity indices, e.g., other markers of hypoxaemia, and in the extension examine whether differences in the access to healthcare might influence which obstructive sleep apnea patients we identify or fail to find.

## AUTHOR CONTRIBUTION

Initial study design: LG, MU; study protocol: JH, MU, LG; data sampling: all authors; data analysis and first draft of the manuscript: MU; critical review of analysis results and editing of the manuscript: all authors; final approval of the manuscript: all authors.

## CONFLICT OF INTEREST

LG reports no conflict of interest for the submitted work. Outside the submitted work he reports grants from Bayer, Philips Respironics Foundation, Resmed Foundation for the ESADA network, non‐financial support and other from Itamar Medical, Fisher and Paykel, Resmed, Philips, Astra Zeneca, and Breas. He has a patent on sleep apnea therapy licensed. JH reports no conflict of interest for the submitted work. Outside the submitted work he reports grants from Bayer, Philips Respironics Foundation, Resmed Foundation for the ESADA network, non‐financial support and other from Itamar Medical, Resmed, Philips, Astra Zeneca, Somnomed and Breas. He is part owner of two licensed patents related to sleep apnea therapy. MU and OS report no conflicts of interest to disclose.

## Supporting information


**TABLE e1** Justifications for including or excluding variables in the model.

**TABLE e2** Clinical and demographic data. Numerical variables are given as mean ± standard deviation. Categorical variables are given as per cent. Comorbidities refer to the range of the number of comorbid conditions each patient has. The comorbid conditions in the SESAR dataset are heart failure, diabetes, atrial fibrillation, asthma/COPD, depression, coronary heart disease, cerebrovascular disease, and hypertension, although some of these conditions have been added after the inception of the registry. Clinics that have contributed with less than 20 patients are included in the total sample, but are not presented separately in this table.

**TABLE e3** Logistic regression models for excessive daytime sleepiness, using Epworth Sleepiness Scale 15 as the cutoff. Continuous predictors are standardised. Numbers are rounded to three significant digits to improve readability. Values reported as ±0.00 are therefore values with an absolute value less than 0.005. ^1^ The effect of age on sleepiness is stronger in women than in men.

## Data Availability

Data sharing from SESAR is regulated by Swedish law. Research using SESAR data has been carried out following approval by an ethics committee and the data committee from the Centre of Registers Västra Götaland. Please contact the Centre of Registers Västra Götaland in Sweden for more information (registercentrum@vgregion.se). More detailed analysis results are available from the corresponding authors upon reasonable request.
