# Supplementary material for: Correlates of excessive daytime sleepiness in obstructive sleep apnea: Results from the nationwide SESAR cohort including 34,684 patients
Source: J Sleep Res. 2022 Jul 22;31(6):e13690. doi: 10.1111/jsr.13690 (PMC9788005; doi:10.1111/jsr.13690)
Supplement: Supplementary file 1 — TABLE e1 Justifications for including or excluding variables in the model. [file JSR-31-e13690-s002.docx]

**Table e1: Justifications for including or excluding variables in the model**

| **Variable** | **Reason** |
| --- | --- |
| Centre | Theoretically justified as referral practices may depend on local traditions and availability of respiratory polygraphy. |
| Age | Included due to previous research (Budhiraja et al 2017) as well as univariate significance. |
| Gender | Included due to previous research (Chervin et al 2000) as well as univariate significance. |
| ODI | Included instead of AHI, as AIC and BIC indicated better model fit if ODI was chosen instead of AHI as a measure of disease severity. |
| AHI | Excluded, as including it together with ODI caused regression model diagnostics to indicate multicollinearity with ODI (i.e., an unacceptably high variance inflation factor), due to them being very strongly correlated. |
| Average O2 saturation | Included due to previous research (Mediano et al 2007) as well as univariate significance. |
| BMI | Included due to previous research (Slater et al 2013) as well as univariate significance. |
| Hypertension | Included due to previous research (Martynowicz et al 2017) as well as univariate significance. |
| Coronary heart disease | Included due to previous research (Feng et al 2012) as well as univariate significance. |
| Cerebrovascular disease | Included due to previous research (Feng et al 2012) as well as univariate significance. |
| Atrial fibrillation | Included due to significant association in univariate models. |
| Depression | Included due to previous research (eg Bixler et al 2005, Budhiraja et al 2017) as well as univariate significance. |
| Diabetes | Included due to previous research (eg Bixler et al 2005) |
| Heart failure | Included due to previous research (patients with comorbid heart failure and OSA are not likely to experience excessive daytime sleepiness, at least according to some research; se Pak et al 2019). |
| Asthma/COPD | Included due to previous research (Kapur et al 2005). |
| Type of diagnostic test (e.g., polysomnography or respiratory polygraphy) | Not included, as polysomnography in Sweden is typically only done in special cases (e.g., prior to multiple sleep latency test). OSA patients diagnosed through PSG are therefore few and likely to differ with regard to a number of other potentially confounding factors. |
| Smoking | Included due to previous research (Wang et al 2021) as well as univariate significance. |

**References**

Mediano, O., Barcelo, A., de la Pena, M., Gozal, D., Agusti, A., & Barbe, F. (2007). Daytime sleepiness and polysomnographic variables in sleep apnoea patients. European Respiratory Journal, 30, 110–113. <https://doi.org/10.1183/09031936.00009506>

Wang, X., Li, W., Zhou, J., Wei, Z., Li, X., Xu, J., Zhang, F., & Wang, W. (2021). Smoking and sleep apnea duration mediated the sex difference in daytime sleepiness in OSA patients. Sleep and Breathing, 25, 289–297.
