# Supplementary material for: Correlates of excessive daytime sleepiness in obstructive sleep apnea: Results from the nationwide SESAR cohort including 34,684 patients
Source: J Sleep Res. 2022 Jul 22;31(6):e13690. doi: 10.1111/jsr.13690 (PMC9788005; doi:10.1111/jsr.13690)
Supplement: Supplementary file 2 — TABLE e2 Clinical and demographic data. Numerical variables are given as mean ± standard deviation. Categorical variables are given as per cent. Comorbidities refer to the range of the number of comorbid conditions each patient has. The comorbid conditions in the SESAR dataset are heart failure, diabetes, atrial fibrillation, asthma/COPD, depression, coronary heart disease, cerebrovascular disease, and hypertension, although some of these conditions have been added after the inception of the registry. Clinics that have contributed with less than 20 patients are included in the total sample, but are not presented separately in this table. [file JSR-31-e13690-s001.docx]

| Centre | N | Age (years)  M±SD | Gender (%)(M/F) | ESS  M±SD | AHI (h^-1^)  M±SD | ODI (h^-1^)  M±SD | Comorbidities (see legend) | Currently smoking (%) | BMI (kg·m^2^)  M±SD |
| --- | --- | --- | --- | --- | --- | --- | --- | --- | --- |
| Total sample | 34,684 | 55.7±13.7 | 67.2/32.9 | 9.7±4.9 | 29.1±22.3 | 24.9±21.4 | 0-8 | 14.4 | 30.2±6.3 |
| Avesta 1 | 2884 | 56.6±13.0 | 65.0/35.0 | 8.5±4.5 | 27.0±22.5 | 24.3±21.8 | 0-5 | 12.2 | 31.4±6.8 |
| Avesta 2 | 318 | 56.4±14 | 47.5/52.5 | No data | 17.5±18.6 | 16.0±17.2 | 0-2 | 12.5 | 29.4±5.5 |
| Borås | 2377 | 57.5±12.8 | 69.0/31.0 | 10±5.1 | 33.7±23.0 | 29.8±22.4 | 0-6 | 13.5 | 30.9±6.0 |
| Eksjö | 1062 | 54±13.2 | 67.6/32.4 | 10.2±4.8 | 28.2±22.6 | 26.3±22.3 | 0-4 | 12.6 | 30.6±5.9 |
| Gothenburg 1 | 1228 | 56.1±13.9 | 64.6/35.4 | 9.2±5.0 | 23.1±19.6 | 19.5±18.4 | 0-5 | 9.3 | 28.6±5.0 |
| Gothenburg 2 | 2348 | 56.5±13.6 | 67.0/33.0 | 10.6±5.2 | 22.7±19.7 | 22.4±20.5 | 0-5 | 17.2 | 29.8±6.0 |
| Gothenburg 3 | 1317 | 56.1±13.2 | 69.3/30.7 | 10.6±4.7 | 32.8±20.2 | 26.8±19.6 | 0-4 | 18.4 | 30.0±5.4 |
| Halmstad | 1665 | 58.1±12.7 | 70.9/29.1 | 9.6±5.0 | 35.6±23.0 | 32.4±22.0 | 0-4 | 11.2 | 30.2±6.1 |
| Jönköping | 1957 | 50.7±15.0 | 65.3/34.7 | 9.5±4.7 | 22.3±22.2 | 21.8±21.9 | 0-8 | 14.0 | 29.2±6.0 |
| Kungsbacka | 1241 | 56.7±13.7 | 67.5/32.5 | 8.8±4.9 | 23.4±19.5 | 21.9±18.7 | 0-4 | 10.0 | 28.3±5.0 |
| Lidköping | 1830 | 57.0±13.5 | 68.4/31.6 | 10.4±4.7 | 31.8±20.2 | 25.4±20.5 | 0-5 | 10.6 | 30.4±5.9 |
| Linköping | 462 | 58.9±13.0 | 69.9/30.1 | 9.9±5.1 | 36.7±22.7 | 34.7±22.1 | 0-5 | 10.0 | 31.7±6.8 |
| Lund | 590 | 54.8±13.7 | 67.6/32.4 | 9.3±4.9 | 22.2±18.2 | 21.9±18.1 | No data | No data | 29.4±5.2 |
| Mölndal | 47 | 58.1±14 | 72.3/27.7 | 11.1±4.6 | 30.0±18.7 | 27.1±18.3 | 0-4 | 31.9 | 29.2±5.1 |
| Norrtälje | 656 | 56.5±15.1 | 64.6/35.4 | 8.6±4.6 | 25.0±22.9 | 23.0±21.4 | 0-5 | 17.9 | 30.2±6.4 |
| Skövde | 3081 | 56.5±13.7 | 67.4/32.6 | 10.9±4.4 | 32.6±20.7 | 25.5±20.8 | 0-6 | 15.9 | 30.8±5.9 |
| Stockholm 1 | 6276 | 55.2±12.9 | 69.0/31.0 | 8.9±5.0 | 33.2±22.7 | 23.2±20.8 | 0-5 | 19.0 | 30.0±7.1 |
| Stockholm 2 | 795 | 56.1±14.4 | 66.9/33.1 | 8.7±5.1 | 20.1±19.2 | 18.6±18.6 | 0-0 | 13.8 | 29.0±5.5 |
| Stockholm 3 | 68 | 42.7±12.5 | 79.4/20.6 | 11.3±6.9 | 30.3±19.8 | 24.6±20.7 | 0-0 | 42.9 | 30.8±12.2 |
| Värnamo | 1189 | 54.9±13.7 | 67.3/32.7 | 10.2±4.8 | 34.2±25.2 | 32.4±24.3 | 0-5 | 14.1 | 30.4±5.9 |
| Västerås | 826 | 57±13.8 | 69.4/30.6 | 10±5.1 | 37.3±23.2 | 35.4±22.9 | 0-6 | 12.1 | 32.7±6.5 |
| Ystad | 203 | 58.2±12.6 | 70.9/29.1 | 9.5±4.5 | 30.4±23.5 | 29.7±22.6 | 0-4 | 20.0 | 31.1±5.3 |
| Örebro | 2245 | 52.7±14.7 | 61.4/38.6 | 10.8±4.9 | 22.1±21.0 | 20.8±20.2 | 0-8 | 11.4 | 30.2±6.3 |

Table e2: Clinical and demographic data. Numerical variables are given as mean±standard deviation. Categorical variables are given as per cent. Comorbidities refer to the range of the number of comorbid conditions each patient has. The comorbid conditions in the SESAR dataset are heart failure, diabetes, atrial fibrillation, asthma/COPD, depression, coronary heart disease, cerebrovascular disease, hypertension and hyperlipidemia, although some of these conditions have been added after the inception of the registry in 2011. Clinics that have contributed with less than 20 patients are included in the total sample, but are not presented separately in this table.
