# Supplementary material for: Correlates of excessive daytime sleepiness in obstructive sleep apnea: Results from the nationwide SESAR cohort including 34,684 patients
Source: J Sleep Res. 2022 Jul 22;31(6):e13690. doi: 10.1111/jsr.13690 (PMC9788005; doi:10.1111/jsr.13690)
Supplement: Supplementary file 3 — TABLE e3 Logistic regression models for excessive daytime sleepiness, using Epworth Sleepiness Scale 15 as the cutoff. Continuous predictors are standardised. Numbers are rounded to three significant digits to improve readability. Values reported as ±0.00 are therefore values with an absolute value less than 0.005. 1 The effect of age on sleepiness is stronger in women than in men. [file JSR-31-e13690-s003.docx]

|  | **Fully pooled model** | | | **Multilevel model (complete cases)** | | | **Multiply imputed multilevel model** | | |
| --- | --- | --- | --- | --- | --- | --- | --- | --- | --- |
|  | Coefficient estimate (OR) | Standard Error | OR 95% CI | Coefficient estimate (OR) | Standard Error | OR 95% CI | Coefficient estimate (OR) | Standard Error | OR 95% CI |
| Male gender | **-1.12 (0.33)** | **0.22** | **0.21-0.51** | **-0.29 (0.75)** | **0.06** | **0.67-0.84** | **-0.25 (0.78)** | **0.04** | **0.73-0.83** |
| Age | **-0.03 (0.97)** | **0.00** | **0.97-0.98** | **-0.37 (0.69)** | **0.05** | **0.63-0.76** | **-0.33 (0.72)** | **0.03** | **0.68-0.76** |
| ODI | **0.014 (1.01)** | **0.00** | **1.01-1.02** | **0.29 (1.34)** | **0.03** | **1.26-1.42** | **0.24 (1.28)** | **0.02** | **1.23-1.32** |
| Average saturation | -0.01 (0.99) | 0.01 | 0.97-1.02 | -0.03 (0.97) | 0.03 | 0.91-1.04 | **-0.06 (0.94)** | **0.02** | **0.91-0.98** |
| BMI | -0.00 (1.00) | 0.00 | 0.99-1.00 | -0.04 (0.97) | 0.03 | 0.91-1.02 | -0.00 (1.00) | 0.02 | 0.96-1.03 |
| Hypertension | -0.08 (0.92) | 0.06 | 0.82-1.04 | -0.08 (0.92) | 0.06 | 0.82-1.04 | **-0.20 (0.82)** | **0.04** | **0.76-0.89** |
| Coronary Heart Disease | -0.01 (0.99) | 0.11 | 0.79-1.24 | -0.02 (0.98) | 0.11 | 0.78-1.23 | -0.10 (0.91) | 0.07 | 0.80-1.03 |
| Cerebrovascular Disease | 0.05 (1.05) | 0.14 | 0.79-1.39 | 0.07 (1.08) | 0.14 | 0.80-1.42 | -0.04 (0.96) | 0.08 | 0.81-1.14 |
| Atrial Fibrillation | -0.13 (0.88) | 0.11 | 0.70-1.10 | -0.17 (0.85) | 0.12 | 0.67-1.06 | **-0.25 (0.78)** | **0.07** | **0.67-0.90** |
| Depression | **0.31 (1.36)** | **0.07** | **1.18-1.58** | **0.28 (1.32)** | **0.07** | **1.14-1.53** | **0.20 (1.23)** | **0.04** | **1.12-1.34** |
| Heart Failure | -0.06 (0.94) | 0.17 | 0.67-1.29 | -0.09 (0.92) | 0.17 | 0.66-1.26 | 0.01 (1.01) | 0.09 | 0.84-1.21 |
| Diabetes | 0.08 (1.09) | 0.09 | 0.92-1.28 | 0.07 (1.07) | 0.09 | 0.91-1.27 | -0.00 (1.00) | 0.05 | 0.90-1.10 |
| COPD/Asthma | 0.07 (1.08) | 0.08 | 0.91-1.27 | 0.04 (1.04) | 0.08 | 0.88-1.22 | 0.07 (1.07) | 0.07 | 0.92-1.24 |
| Daily smoking | 0.13 (1.14) | 0.08 | 0.97-1.34 | 0.13 (1.14) | 0.08 | 0.97-1.35 | **0.12 (1.13)** | **0.05** | **1.02-1.24** |
| Gender × Age^1^ | **0.01 (1.01)** | **0.00** | **1.01-1.02** | **0.20 (1.22)** | **0.05** | **1.09-1.36** | **0.15 (1.16)** | **0.03** | **1.09-1.24** |

Table e3. Logistic regression models for excessive daytime sleepiness, using ESS 15 as cutoff. Continuous predictors are standardised. Numbers are rounded to three significant digits to improve readability. Values reported as ±0.00 are therefore values with an absolute value less than 0.005. ^1^ The effect of age on sleepiness is stronger in women than in men.

Table e3. Logistic regression coefficients with excessive daytime sleepiness defined as ESS≥15, presented as odds ratios with 95% confidence intervals.
